# O6-7 Community sport coach's potential to enhance health

**DOI:** 10.1093/eurpub/ckac094.047

**Published:** 2022-08-29

**Authors:** Wikke van Stam, Ine Pulles, Caroline van Lindert, Eva Heijnen

**Affiliations:** Mulier Instituut, Utrecht, The Netherlands

**Keywords:** Community sport coach, Local sport policy instrument, Local implementation

## Abstract

In 2008 Community sport coaches (CSC) were introduced in the Netherlands as a policy instrument to motivate inhabitants to become physically active, by connecting the sport sector with other sectors (e.g. health, education). Almost 6000 CSC are currently active throughout 98% of Dutch municipalities. We used Jolley's model to examine the use of CSC as a complex local sport policy instrument for health enhancement (Jolley, 2014; Van Lindert et al., 2017). The question we aimed to answer was: To which extent is the CSC program used for health enhancement? We will share the impact of this policy program in the Netherlands and discuss the relevance and implications for other countries.

In 2019, we conducted an online questionnaire to examine the health incentives and outcomes (e.g. sectors, target groups, goals) of local CSC programmes among all municipalities implementing CSC (n = 347). We also surveyed our CSC (n = 100) and CSC employers (n = 27) panels about their work on health enhancement.

Findings show that health enhancement is not often an explicit goal of CSC programmes. No national goals of the program address health explicitly and only 27% of municipal goals for CSC are focused on health. Nevertheless, the governmental health department and/or local health parties are often (82%) involved in CSC policy decision making (e.g. by paying part of the finances and/or by employing CSC). This results in a contribution to health enhancement of almost all (98%) local CSC programmes. Findings show that CSC connect the sport sector with the health sector in 96% of the municipalities, coaches/employers have often collaborated with health organisations last year (respectively 81% and 100%) and/or CSC work with inactive and/or overweighed inhabitants (69%)).

CSC are responsible for motivating people to become physically active, and logically this has an impact on health. Despite the lack of explicit goals, local CSC programmes are intertwined with health incentives and outcomes. But often the exact effects on health are unknown. More explicit and specific health goals may increase the impact of sport policy on health enhancement. Facilitators and barriers relevant to further national and international implementation of CSC programmes will be discussed.

